# A drug screening assay on cancer cells chronically adapted to acidosis

**DOI:** 10.1186/s12935-018-0645-5

**Published:** 2018-09-25

**Authors:** Paola Pellegrini, Jason T. Serviss, Thomas Lundbäck, Nicolo Bancaro, Magdalena Mazurkiewicz, Iryna Kolosenko, Di Yu, Martin Haraldsson, Padraig D’Arcy, Stig Linder, Angelo De Milito

**Affiliations:** 1 Cancer Center Karolinska, R8:00, Department of Oncology-Pathology, Karolinska Institute, 171 76 Stockholm, Sweden; 2 Chemical Biology Consortium Sweden, Science for Life Laboratory, Stockholm, Sweden; 3 Department of Medical and Health Sciences, Linköping University, 581 83 Linköping, Sweden; 4 Present Address: Discovery Sciences, IMED Biotech Unit, AstraZeneca, Gothenburg, Sweden

**Keywords:** Tumor acidosis, Drug resistance, RNAseq, Verteporfin, Drug screening

## Abstract

**Background:**

Drug screening for the identification of compounds with anticancer activity is commonly performed using cell lines cultured under normal oxygen pressure and physiological pH. However, solid tumors are characterized by a microenvironment with limited access to nutrients, reduced oxygen supply and acidosis. Tumor hypoxia and acidosis have been identified as important drivers of malignant progression and contribute to multicellular resistance to different forms of therapy. Tumor acidosis represents an important mechanism mediating drug resistance thus the identification of drugs active on acid-adapted cells may improve the efficacy of cancer therapy.

**Methods:**

Here, we characterized human colon carcinoma cells (HCT116) chronically adapted to grow at pH 6.8 and used them to screen the Prestwick drug library for cytotoxic compounds. Analysis of gene expression profiles in parental and low pH-adapted cells showed several differences relating to cell cycle, metabolism and autophagy.

**Results:**

The screen led to the identification of several compounds which were further selected for their preferential cytotoxicity towards acid-adapted cells. Amongst 11 confirmed hits, we primarily focused our investigation on the benzoporphyrin derivative Verteporfin (VP). VP significantly reduced viability in low pH-adapted HCT116 cells as compared to parental HCT116 cells and normal immortalized epithelial cells. The cytotoxic activity of VP was enhanced by light activation and acidic pH culture conditions, likely via increased acid-dependent drug uptake. VP displayed the unique property to cause light-dependent cross-linking of proteins and resulted in accumulation of polyubiquitinated proteins without inducing inhibition of the proteasome.

**Conclusions:**

Our study provides an example and a tool to identify anticancer drugs targeting acid-adapted cancer cells.

**Electronic supplementary material:**

The online version of this article (10.1186/s12935-018-0645-5) contains supplementary material, which is available to authorized users.

## Background

Tumor cells within a tissue are constantly under the selective pressure of the surrounding environment, characterized by heterogeneity in terms of cellular components, availability of nutrients, fluctuating oxygen levels, poor tissue perfusion and low pH [1, 2]. Such a complex environment is poorly reproduced by standard in vitro culture conditions, which provide excessive levels of nutrients and growth factors, an atmospheric oxygen pressure never reached in human tissues (21%) and the physiological pH conditions of healthy tissues (pH 7.4). Drug-resistance is one of the most important problems in clinical oncology [3], yet anticancer drugs are normally identified and tested using screening procedures that do not consider the biochemical and physical complexity of solid tumors [4]. This classical approach usually results in the identification of substances that inhibit cell proliferation, but not necessarily affecting the survival of non-proliferating cancer cells. One potential approach to overcome multicellular resistance is the use of multicellular spheroids (MCS), since this 3D culture system better reproduces the oxygen, nutrients and pH gradients observed in human tumor tissues [5, 6]. Results from different studies have indicated that such model systems reduce the chances of selecting anticancer compounds with insufficient pharmacological properties.

One of the simplest biochemical properties affecting drugs’ activity, distribution and intracellular accumulation is their charge status in different pH conditions [7, 8]. Cells within solid tumors are known to be exposed to chronic and/or intermittent acidic conditions as a consequence of high metabolic rates, producing acid metabolites which are not sufficiently removed because of scarce vascular perfusion [1, 9]. Acidification of the extracellular environment causes cell cycle arrest, decreased glycolysis and protein synthesis, autophagy and increased angiogenesis and metastases [8, 10]. Besides promoting malignant progression of tumors, tumor acidosis represents an important mechanism of drug resistance, likely associated with poor drug efficacy [3, 8, 11, 12]. In an attempt to overcome acidosis-mediated drug resistance, we developed a drug screening assay to target the colon cancer HCT116 cell line chronically adapted to acidosis. The screening of the Prestwick library compounds led to the identification of the photosensitizer Verteporfin (VP) as an effective compound with preferential activity towards cells in acidic conditions. We also discovered several aspects of the Verteporfin mechanism of action that raise several cautions with regards to interpretation of experimental results with this drug.

## Materials and methods

### Chemical and antibodies

RPMI-1640 (SH30255.01), trypsin (SH40003.12), phosphate-buffered saline (PBS, SH40003.12) and Fetal Bovine Serum (FBS) (SV30160.03) were purchased from HyClone. Sodium Bicarbonate (25080) and RPMI-1640 without NaHCO_3_ (51800) were purchased from Gibco. Bafilomycin A1 (BafA1, B1793), protease cocktail tablets EDTA-free, phosphatase inhibitors (P5726, P0044) and Verteporfin were purchased from Sigma. Protein assay dye reagent concentrate (500-0006), protein assay standard (5000-0007) and dry milk (170-6404) were purchased from Bio-Rad. The following antibodies were used: LC3B (Cell Signaling Technology, 2775), SQTM1/p62 (BD Biosciences, 610833) and β-actin (Sigma, A5441). HRP-conjugated anti-rabbit (NA934V) and anti-mouse (NXA931) antibodies, ECL system (RPN2106) and PVDF membranes (RPN303F) were purchased from GE Healthcare. The fluorogenic substrate Suc-LLVY-AMC (S-280) was purchased from Boston Biochem (USA).

### Cell culture

The colon carcinoma cell line HCT116 was cultured in RPMI-1640 medium supplemented with 10% FBS, antibiotics and 2 g/l NaHCO_3_. The low pH adapted HCT-116 cell line (AA-HCT116) was obtained by growing the parental cells in RPMI-1640 medium buffered at pH 6.8 (0.15 g/l NaHCO_3_) for 3 months as previously described [13, 14]. In the settings used, media pH was stable over 48 h and dropped by about 0.1–0.2 units after 3 days in culture for cells kept at pH 6.8 and by 0.3–0.4 units for parental cells. Since parental cells have a higher proliferation rate, so was medium pH dropping faster and cells were passaged every 2–3 days. AA-HCT116 cells were passaged every 4–5 days. The h-TERT RPE1 cell line was cultured in DMEM F12 medium supplemented with 10% FBS and antibiotics. The MelJuSo UbG76V-YFP cell line was cultured in DMEM medium completed with 10% FBS and antibiotics.

### Measurements of extracellular acidification rate (ECAR) and oxygen consumption rate (OCR)

HCT116 cells were plated at 60,000 cells/well in 100 µl medium in XF24-well cell plates. Subsequently medium was replaced with 500 µl Seahorse assay medium (1 mM pyruvate, 25 mM glucose and 2 mM glutamine). Oligomycin, FCCP, rotenone and antimycin A were injected according to the Seahorse XF cell mito stress kit. The mitochondrial function was assessed detecting the ECAR and OCR values by using the Seahorse XF analyzer.

### Drug screening assay

AA-HCT116 cells were plated at 15,000 cells/well in flat-bottom 96-wells plate, left under the laminar hood for 1 h and then moved to the incubator. On the next day, all plates were treated simultaneously with 10 µM of each of the compounds from the Prestwick chemical library (one compound per well). For each plate, quadruplicate wells were used as untreated controls and quadruplicate wells used for the positive control (VLX600 10 µM) [15]. Plates were then moved back to the incubator and 48 h later cell viability was assessed by the acid phosphatase assay [16]. Hits were identified using a threshold of 70% inhibition of cell viability. Hit confirmation was performed testing each compound in a dose range 0–15 µM in duplicate wells.

### Cell viability and clonogenic assay in monolayer and multicellular spheroids cultures

Cells were plated in 96-well plates and the next day treated with different concentrations of the specific compounds. Forty-eight hours after treatment cell viability was evaluated by using the acid phosphatase assay.

For analysis of clonogenic cell survival, HCT116 and AA-HCT116 cells were plated at 300 cells/well in 6-well plates and treated with VP for 8 or 48 h, following culture for 6–8 days in drug-free media. Plates were then washed in PBS and colonies were stained with Giemsa.

Clonogenic cell survival was also assessed on HCT116 MCS treated with different compounds. Briefly, MCS were obtained by a modification of the hanging-drop method applied to 96-well plates as previously described [17]. After drug treatment for 3 days, the MCS were collected, washed in PBS, and a single cell suspension was obtained after incubation with Accutase (Biolegend, 423201). Equal volumes of cells for each treatment condition were plated in triplicate in 6-well plates for 6–8 days, followed by staining the colonies with Giemsa. The software ImageJ was used to perform semi-automated colony counting.

### Analysis of intracellular VP accumulation

Cells were plated in black 96-wells plates (Costar) and allowed to adhere overnight.

On the next day, VP was added in a range 0–15 μM for 4 h. After removal of drug containing medium, cells were extensively washed with PBS. VP fluorescence was recorded with excitation wavelength of 450 nm and an emission wavelength of 695 nm [18] using a fluorescence microplate reader Tecan Infinite M1000 (Tecan, Männedorf, Switzerland). For each condition quadruplicate wells were used and VP fluorescence was normalized to protein content for each well.

Qualitative analysis of intracellular VP was performed by laser-scanning fluorescence microscopy with a Leica confocal microscope.

### Studies on autophagic flux

Analysis of the autophagic flux by Western blot was done on different cell lines. Experiments were performed by plating cells in their appropriate medium and allowing them to adhere overnight. On the next day, VP was added at specified concentrations for 4, 8 or 24 h and BafA1 (100 nM) was added during the last 2 h of incubation. Cells were then collected for Western blotting. Analysis of the autophagic flux was performed using the ratio of normalised levels of LC3-II in the presence and absence of BafA1. All experiments were performed in darkness conditions.

### Western blotting

Cells were washed with PBS on ice and collected by scraping in cold PBS. The cell pellet was lysed in RIPA buffer (150 mM NaCl, 50 mM Tris pH 7.4, 1% Nonidet P-40, 0.1% SDS and 0.5% sodium deoxycholate) in presence of protease and phosphatase inhibitors. The protein concentration was determined using the Biorad Protein Assay (Biorad Laboratories) and equal amount of proteins (20 µg) was loaded on pre-casted acrylamide gels (4–12% SDS-PAGE, NuPage). The proteins were transferred from the gel to PVDF membrane for 2 h at 4 °C. Red Ponceau staining of the membranes verified the proper loading and transfer. Membranes were blocked in 5% blotting grade dry milk in TBS with 0.1% Tween (TBS-T) for 1 h at room temperature and then incubated with primary antibodies diluted in 5% BSA in TBS-T overnight at 4 °C. The next day membranes were washed and incubated for 1 h at room temperature with the appropriate HRP-conjugated secondary antibody and the binding was revealed by the ECL system.

### Analysis of proteasomal activity

The fluorogenic substrate Suc-LLVY-AMC was used to measure the chymotrypsin-like activity of the proteasome on cellular lysates. Briefly, cells were treated with VP or BZ for 4 h, lysed in presence of 0.05% NP40, 1 µM DTT and 2 µM ATP, followed by the addition of Suc-LLVY-AMC (10 μM). Fluorescence intensity was recorded using a Tecan Infinite M1000 microplate reader with excitation wavelength 380 nm and emission wavelength 460 nm.

The cell line MelJuSo UbG76 V-YFP was used as reporter of proteasome inhibition [19]. Cells were plated overnight and then exposed to VP or BZ. Detection of accumulated Ub-YFP was performed by Western blot analysis using an anti-GFP antibody.

### RNA sequencing and bioinformatics

RNA sequencing was performed in biological triplicate using Strand-specific TruSeq library preparation and Ribo-Zero ribosomal depletion. Tophat2, HTSeq, and DESeq 2 were utilized for alignment, quantification, and differential expression analysis, respectively, with the hg19 genome and Ensembl v73 [20–22]. All bioinformatics were preformed in the R programming language [23] and scripts related to the differential expression analysis, gene ontology analysis, and community detection analysis are publically available (https://github.com/GranderLab/acidAdaptedRNAseq) as an R package facilitating reproduction of the analysis and associated figures. Principal component analysis and hierarchical clustering was performed using the prcomp and heatmap.2 functions, respectively, within the R statistical environment. Data was centered and scaled (mean = 0, sd = 1) for each gene previous to clustering and regularize log transformed previous to PCA.

### Gene ontology term enrichment

Significant (alpha < 0.05) genes in the differential expression analysis were used for biological processes GO term enrichment analysis with the topGO software [24]. All quantified genes, defined as counts per million > 1 in at least 3 samples, were utilized as the gene universe. Terms with less than 5 annotated genes were not included in the significance testing procedure. Significance testing was performed using the classic Fisher method and the top 1000 terms with the lowest p-value were included in downstream analysis unless otherwise specified.

### Community detection analysis

To understand the relationship between the significant GO terms, the GO graph was retrieved for all significant (alpha < 0.05) GO terms using the GOSim Bioconductor package [25, 26]. The GO graph was then utilized for community detection via the spin-glass algorithm [27] from the igraph package [28] using a maximum of 200 possible spin states. The authority score was subsequently calculated for all nodes in the GO graph to determine the node with the highest in-degree for each community [29]. The GO graph was then collapsed on the nodes with the highest authority scores (community node) by merging all nodes into said node and simplifying the graphs edges. In cases when several nodes had identical authority scores equaling the max authority score for that community’s nodes, one was chosen at random to represent the community. All nodes not presently contained in the collapsed graph, were merged into the graph and edges were re-drawn between the merged nodes and the community node. Results were visualized with the graph package [30].

### Statistical analysis

All data were obtained from at least three experimental replicates and presented as mean ± SEM, if not otherwise indicated. Data analysis was performed using Graphpad Prism 6.0 (GraphPad Software, CA). Differences between groups were analysed with parametric or non-parametric tests according to the distribution of the values. The specific test used is indicated in figure legends. The significance level was set as P < 0.05.

## Results

### Acid-adapted HCT116 cells are resistant towards many clinically used drugs

**Fig. 1:**
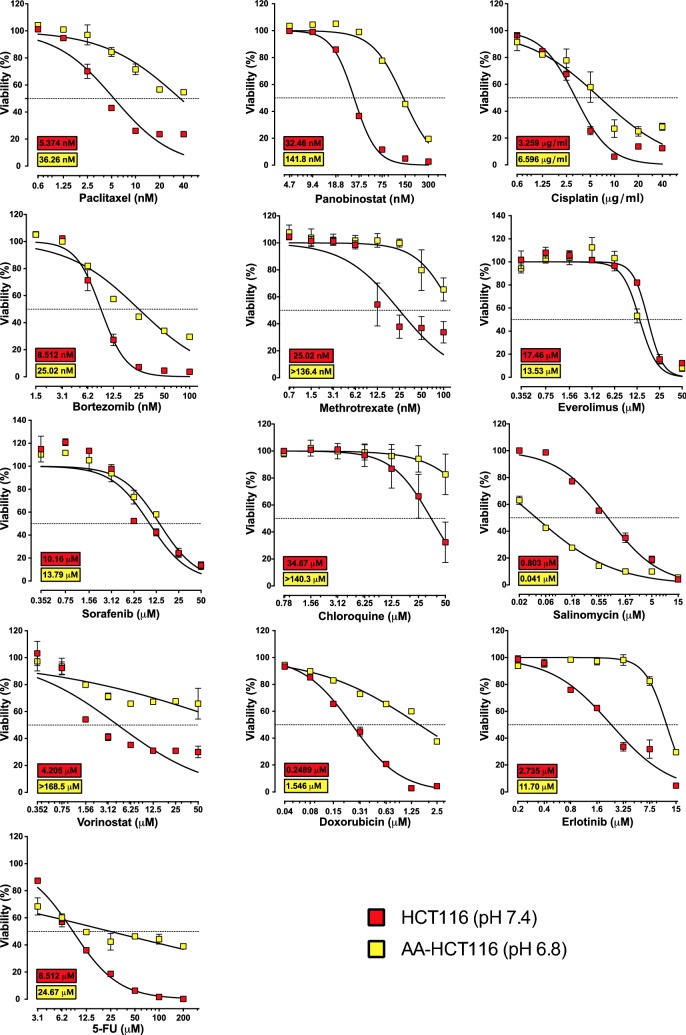
Effect of acidosis on cell viability. HCT116 (red boxes) and AA-HCT116 (yellow boxes) cells were treated with different anticancer drugs and viability was measured after 3 days. The IC_50_ for each drug and condition is shown in the respective boxes. Data from at least two experiments are shown

It has been previously shown that cancer cells are less sensitive and/or completely resistant to several clinically used drugs in conditions of acidosis, especially when the compounds are basic [8, 31–33]. To allow studies of such resistance, the colon carcinoma cell line HCT116 was adapted to grow in conditions of chronic acidosis at pH 6.8 starting from the bulk cell population. We refer to this acid-adapted sub-line as AA-HCT116 [13, 14]. We then performed a small validation screen to test the respective sensitivities of HCT116 and AA-HCT116 cells towards a small panel of clinically used drugs. These span multiple oncology related drug classes, including DNA damaging agents (doxorubicin, cisplatin, methotrexate, 5FU), a microtubule stabilizer (Paclitaxel), tyrosine kinase inhibitors (Sorafenib and Erlotinib), a mTOR inhibitor (Everolimus), HDAC inhibitors (Vorinostat and Panobinostat), a proteasome inhibitor (Bortezomib) and an autophagy inhibitor (Chloroquine). As shown in Fig. 1, cells chronically adapted to acidosis are less sensitive to most of the drugs under these conditions, while showing similar level of sensitivity towards Everolimus and Sorafenib. None of the tested drugs were more cytotoxic in conditions of acidosis. In this validation run, we used salinomycin as a positive control since we recently reported that this compound has a preferential cytotoxicity under acidic conditions [14]. These results confirm that acidosis negatively affects the efficacy of anticancer drugs. As reported below, these findings prompted us to use this model system in a broader search for compounds with preferential activity towards acid-adapted cells, but prior to this we initiated a more thorough characterization of the AA-HCT116 cells.

### Phenotypic and transcriptomic profile of acid-adapted cancer cells

**Fig. 2:**
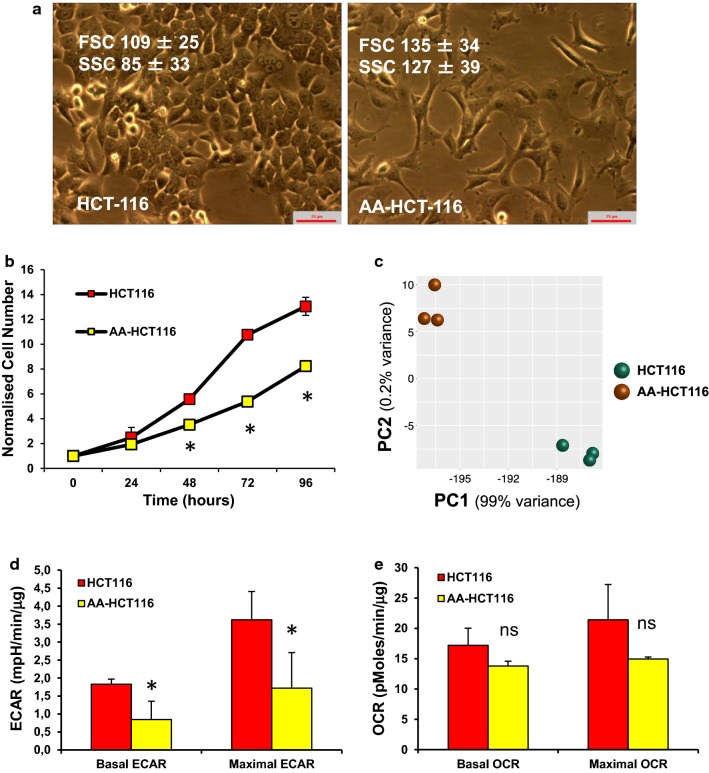
Characterization of acid-adapted HCT116 cells. Morphological (**a**), cell growth (**b**), transcriptomic (**c**) and metabolic (**d**, **e**) characteristics of parental and acid-adapted HCT116 cells. **b** Principal component (PC) analysis performed using the top 500 genes with the most variance in the RNA sequencing data. *P<0.05, *ns* not significant

The adapted AA-HCT116 cells showed dramatic phenotypic changes in comparison to the parental cell line, showing a more mesenchymal-like morphology, a lower proliferation rate (doubling time 17 ± 1.5 vs 23 ± 1.6 h) and increased forward (size) and side scatter (granularity) when analyzed by flow cytometry (Fig. 2a, b). RNA sequencing (RNA-seq) performed on both cell lines showed that AA-HCT116 cells have a unique transcription profile, further indicating that specific molecular changes had taken place during adaptation to low pH conditions (Fig. 2c). Changes to cellular transcriptomes in response to such conditioning have also been reported by others [34]. Analysis of the metabolic profile by using an XF Analyser indicated that AA-HCT116 cells are characterized by a lower basal and oligomycin-induced ECAR, suggesting a decreased glycolytic rate compared to parental cells (Fig. 2d). AA-HCT116 cells also tend to have a lower oxygen consumption rate, although this difference was not statistically significant (Fig. 2e).

We utilized RNA-seq data to perform differential expression analysis and discovered 4796 genes to be significantly (alpha < 0.05) altered in AA-HCT116 cells vs. their parental cell line, with 1283 of these genes exhibiting a fold change > 2 (Additional file 1: Figure S1A).

**Fig. 3:**
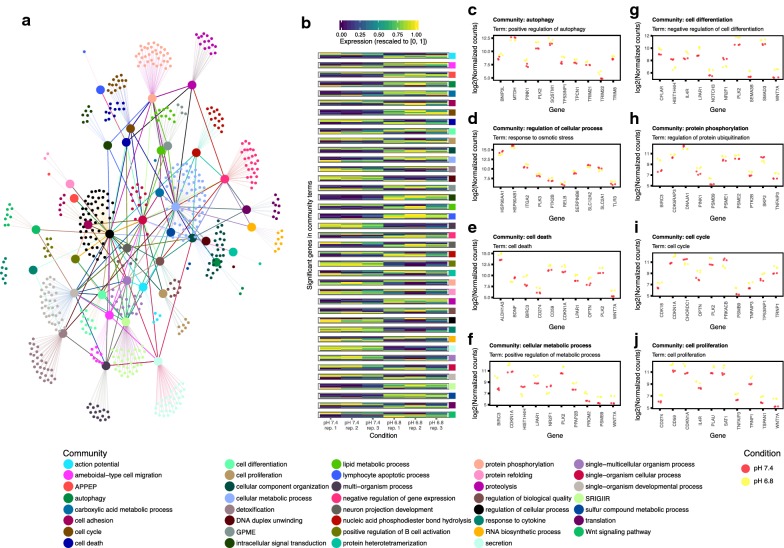
RNAseq data analysis. **a** A network graph visualizing the results from the community analysis. Edge (line) direction is represented by color with edges originating from a node inheriting that nodes color. Edges between community nodes (large points) indicate that the GO terms representing the nodes are each other’s ancestors or offspring dependent on the direction of the edge. Edges between term nodes (small points) and community nodes indicate the terms inclusion in that community. **b** A heatmap representing gene expression profiles in the detected communities. Communities are indicated by the color bar on the right side of the heatmap. Expression scaled to [0, 1] indicates the regularized log transformed expression values scaled between 0 and 1. **c** Gene expression profiles for selected terms in a subset of the detected communities for parental and acid adapted cells. Antigen processing and presentation of exogenous peptide antigen via MHC class I, TAP-independent (APPEP); generation of precursor metabolites and energy (GPME); somatic recombination of immunoglobulin genes involved in immune response (SRIGIIR)

Gene ontology (GO) term enrichment analysis resulted in 579 significantly (alpha < 0.05) enriched terms. Since many of these terms are related to similar biological processes, we desired to summarize these results by identifying groups of similar terms. To achieve this we utilized a community detection algorithm [27] to deduce similar terms within the GO graph using the terms found to be significant, as well as, their ancestors. This resulted in the detection of 39 communities (Fig. 3a). In the communities detected, we identified many terms known to be associated with the biology of acid adapted cells such as cell differentiation, cell death, and cell adhesion, as well as, novel associations such as autophagy, cellular metabolism, and Wnt signaling. Analysis of the gene expression patterns in each community revealed strong contrasts in gene expression between parental and acid adapted cells (Fig. 3b). Finally, analysis of individual expression patterns within each community term revealed both known and novel players in the biology of acid adapted cells (Fig. 3c).

Thus, AA-HCT116 cells maintained under acidic conditions showed characteristics resembling less differentiated cells and with a decreased proliferation rate, further supporting the use of these cells as a model system for identification of compounds targeting cancer cells under these clinically relevant conditions.

### Screening of the Prestwick library of known drugs

**Fig. 4:**
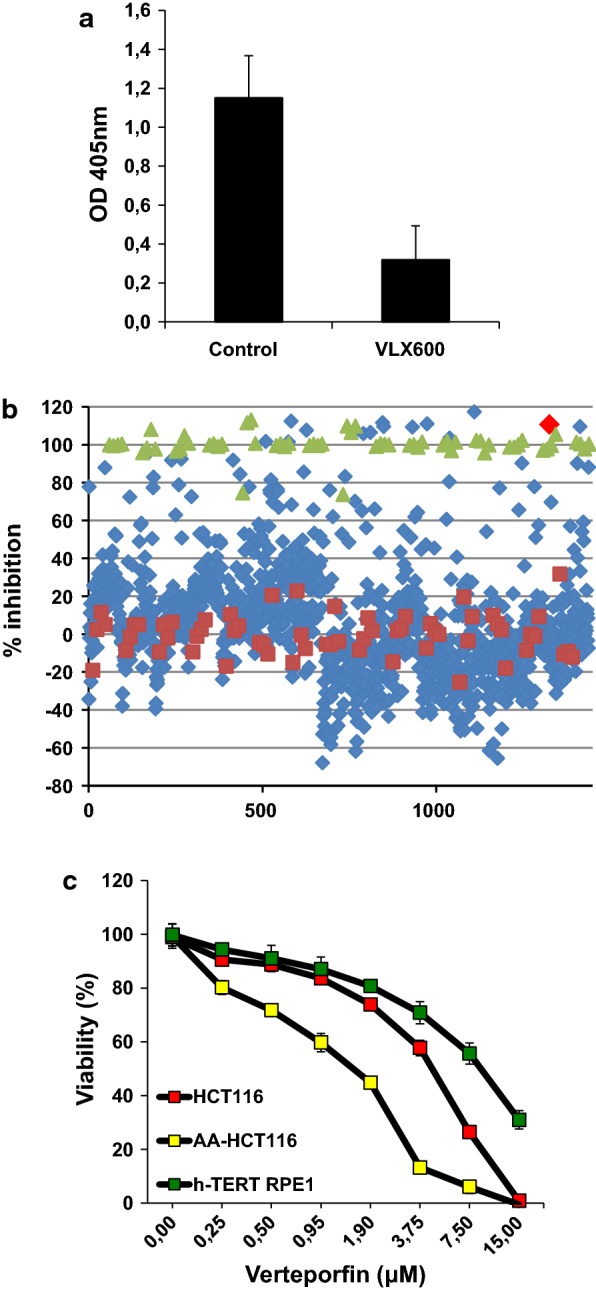
Screening of the Prestwick library. **a** Viability assays showing the killing activity of VLX600 as quality control in the drug screening assay. **b** Percent inhibition of cell viability induced by the library compounds (blue markers) in comparison to the QC (green markers) and DMSO (dark red marker) resulting in 100% and 0% inhibition, respectively. The bright red diamond represents Verteporfin. **c** The cytotoxic activity of VP on HCT116, AA-HCT116 and immortalized hTERT-RPE1 cells is shown

The drug screening assay was applied in a 96-well plate format following the optimization of multiple parameters including FBS concentration and cell densities. In the screening assay the compound VLX600 (10 µM) was used as positive quality control (QC), resulting in about 20% cell viability (Fig. 4a). The Z-factor of the assay was calculated based on controls in each plate and averaged 0.6.

The Prestwick chemical library was applied for our search of cytotoxic compounds with efficacy under acidic conditions. Cells were exposed to 10 µM of each of the 1280 compounds diluted in DMSO for 48 h and cell viability was assessed by acid phosphatase assay. Figure 4b displays the percent inhibition of cell viability observed in the presence of library compounds (blue marker) in comparison to the QC (green marker) and DMSO (dark red marker), which were used to define 100% and 0% inhibition, respectively in data normalization. Hits inducing a loss in cell viability > 70% were selected for confirmation studies in full concentration response assays. Out of the initial 55 hits, 11 compounds were confirmed as having a cytotoxic activity in the range 1–10 µM in both culture conditions (Table 1). To further filter for compounds with what we regarded as a desirable in vitro-based efficacy versus safety profile, we compared the concentration-dependent effects of the 11 hits on cell viability in parental HCT116 cells, AA-HCT116 cells, and in the immortalized epithelial cells, h-TERT RPE1. Among the clinically used compounds, only Verteporfin (Fig. 4b, c) and Auranofin (Additional file 2: Figure S2) showed a small window of decreased toxicity towards normal cells and a tendency for preferential activity towards AA-HCT116 cells. Of the remaining hits from the Prestwick library, some turned out to have detergent-like structures and were thus excluded (Additional file 2: Figure S2). Amongst hits that were not pursued at this stage there are also agents with bactericidal activity, although these could be of interest in follow-up studies.

**Table 1:** Hit compounds from Prestwick compound library

Compound name	% inhibition	Therapeutic group
Chlorhexidine	96	Bacteriostatic
Camptothecin	92	Antitumor agent
Methyl benzethonium chloride	112	Antibacterial
Benzethonium chloride	110	Antibacterial
Thimerosal	66	Antiseptic
Alexidine dihydrochloride	110	Antibacterial
Simvastatin	81	Antihyperlipidemic
Auranofin	111	Antirheumatic
Thonzonium bromide	117	Antiseptic
Pyrvinium pamoate	90	Antiparasitic
Verteporfin	111	Treatment of age-related macular degeneration

### Activity of Verteporfin in acidic and metabolically-stressed colon cancer cells

VP (trade name Visudyne^®^) is a benzoporphyrine derivative used as a photodynamic agent for the treatment of macular degeneration, with limited toxicity. Recent studies have proposed that VP has anticancer activity both in vitro and in preclinical animal models [35, 36]. Moreover, VP has been suggested as a specific inhibitor of the YAP-1 protein whose role is important in the biology of colon carcinoma [37].

**Fig. 5:**
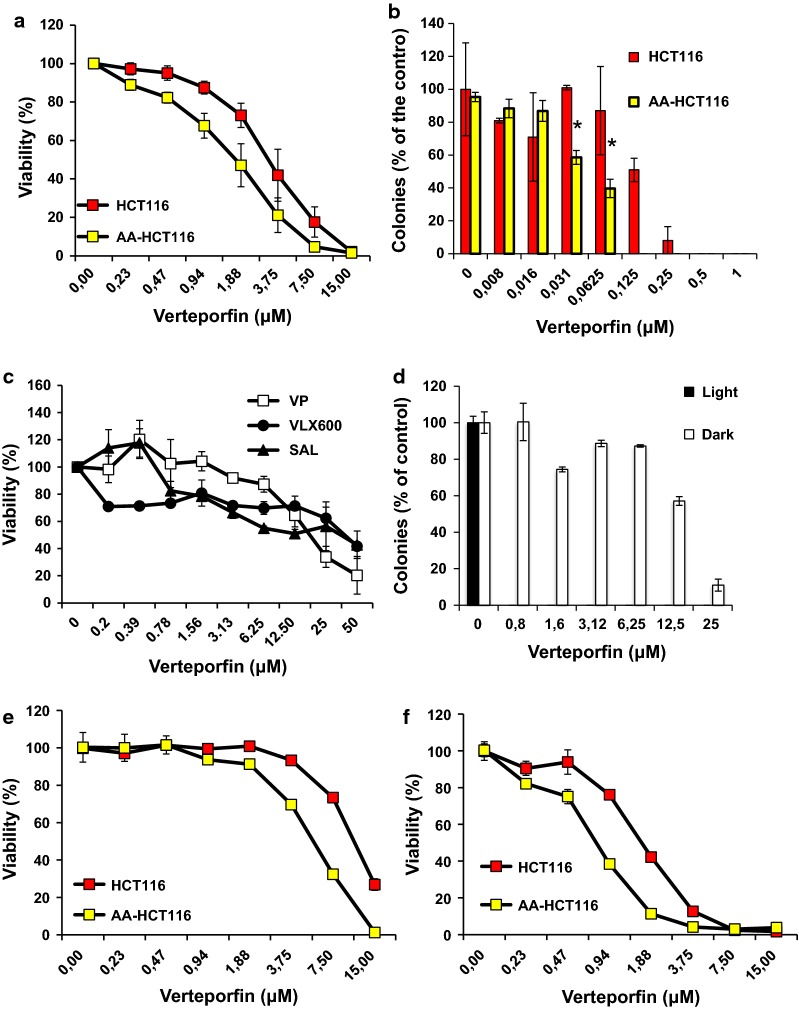
pH and light-dependent activity of VP on cancer cells. Viability assay (**a**) and clonogenic assay (**b**) show that AA-HCT116 are more sensitive than HCT116 cells to VP. Data from viability assay in A were obtained from 5 different experiments and means and SEM are shown. HCT116 MCS are sensitive to the cytotoxic activity of VP as shown by viability assay (**c**) and clonogenic assay (**d**). The cytotoxic activity of VP was tested in conditions of darkness (**e**) and ambient light (**f**) in HCT116 and AA-HCT116 cells. Data from at least two independent experiments are shown

Firstly, we confirmed that VP was equally effective in reducing viability of AA-HCT116 cells with respect to their parental cell line by viability assay (Fig. 5A) and clonogenic cell survival (Fig. 5b) using monolayer cultures. Data from Fig. 5a indicates that the IC_50_ of VP for HCT116 and AA-HCT116 cells are 3.4 ±0 .77 μM and 1.72 ± 0.43 μM, respectively (P≤0.05). Taken together these results suggest that VP can be effective in killing cancer cells in metabolically stressed environments. To confirm this, we tested the activity of VP in the 3D model of multicellular spheroids (MCS). MCS more accurately reproduce the biochemical and metabolic heterogeneity of the tumor environment by containing hypoxic and acidic regions when compared to monolayer cultures [5]. Interestingly, many of the hit compounds identified in our screen have been reported to inhibit mitochondrial function in 3D drug screening models using HCT116 cells suggesting some selectivity for metabolically stressed cores [38]. We observed that VP was effective in reducing cell viability of MCS (Fig. 5c), similar to compounds like salinomycin (SAL) and VLX600 which have been recently reported to have a strong killing activity in MCS [6, 38]. Given the light-sensitive nature of VP we conducted experiments under conditions where the cultures were either expose or not to ambient light. When the activity of VP on MCS was analysed in terms of clonogenic cell survival we observed a dramatic, light-dependent inhibitory effect even at very low VP doses (Fig. 5d). In fact, despite being a photosensitizing agent, VP has been reported to inhibit cell growth of different cancer cell lines even without light activation [39, 40]. In line with these findings, VP exposure to ambient light resulted in a dramatic enhancement in cytotoxic activity in both HCT116 and AA-HCT116 cells (Fig. 5e, f). Importantly, in our experimental settings we did not employ the specific 690 nm wavelength laser light that is routinely used for photodynamic activation of VP, but instead we simply exposed the treated cell cultures to ambient light for 30 min before moving the plates into the incubator.

**Fig. 6:**
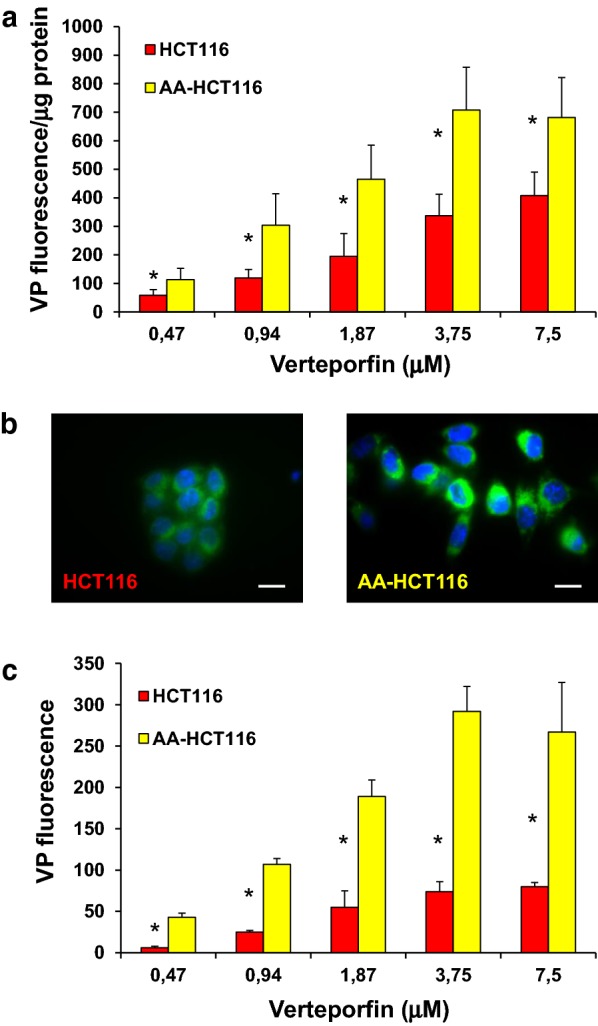
pH-dependent intracellular accumulation of VP. **a** Dose-dependent intracellular accumulation of VP was analysed by fluorimetry in HCT116 and AAHCT116 cells and normalised to protein content. **b** HCT116 and AA-HCT116 cells were treated with VP for 30 min and analysed by confocal microscopy. Scale bar 10 μm. **c** Intracellular accumulation of VP was analysed by fluorimetry in HCT116 cells transiently exposed to medium at pH 7.4 or pH 6.8 for 4 h. *P<0.05

Pharmacological properties of compounds such as solubility, logD and pKa are determinants of drug sensitivity [8, 32]. VP is an acidic compound with a predicted pKa of 4.12 and in line with this we observed increased intracellular VP levels by approximately threefold in AA-HCT116 cells as compared to parental HCT116 cells (Fig. 6a). This increased accumulation in acidic conditions was confirmed by confocal fluorescence microscopy (Fig. 6b) on AA-HCT116 and by fluorimetric analysis on parental HCT116 cells transiently exposed to pH 6.8 (Fig. 6b). These data suggest that acidic conditions enhance uptake and intracellular VP availability.

### VP alters levels of polyubiquitinated proteins without inhibition of the proteasome

**Fig. 7:**
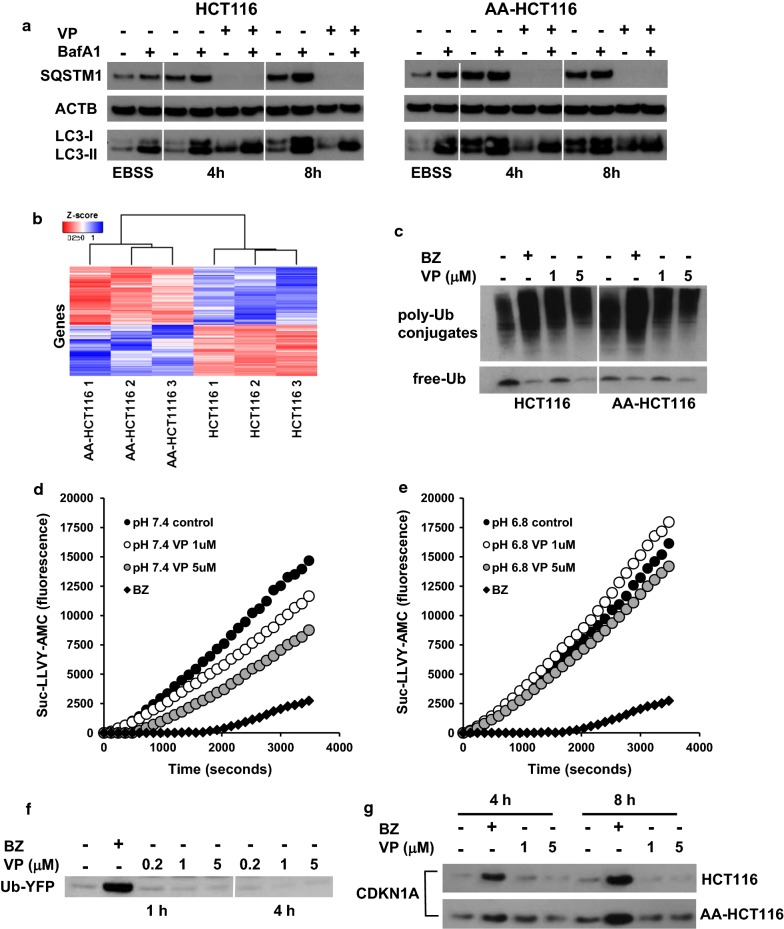
Autophagy and proteasomal activity are not affected by VP. **a** HCT116 and AA-HCT116 cells were exposed to VP for 4 and 8 h, or to EBSS, in presence or absence of BafA1 to analyse the turnover of LC3-II and SQSTM1 by WB. **b** Clustering analysis of the 229 genes associated with the four ubiquitinrelated GO terms found to be significantly enriched in the GO analysis. **c** HCT116 and AA-HCT116 cells were exposed to VP or BZ for 4 h and the presence of poly-Ub conjugates and free Ub was detected by WB. **d**, **e** HCT116 and AAHCT116 cells were treated with VP or BZ for 4 h and cell extracts exposed to the fluorogenic substrate Suc-LLVY-AMC to measure chemotrypsin-like activity of the proteasome. **f** The cell line MelJuSo UbG76 V-YFP was used as reporter of proteosomal inhibition after treatment with VP or BZ. Accumulation of Ub-YFP indicates inhibition of proteasomal activity. **g** The levels of CDKN1A were analysed as marker of proteasomal activity in HCT116 and AA-HCT116 cells treated with BZ or VP at the indicated times and concentrations

Due to the reported activity of VP as an autophagy inhibitor, we next investigated whether it differentially affected autophagic flux (AF) in parental and AA-HCT116 cells. Surprisingly, we observed that AF measured by WB analysis of LC3-II was not significantly altered under VP treatment in either cell line (Fig. 7a). In line with RNAseq data in Fig. 3c, we observe that basal expression of SQSTM1 is increased in AA-HCT116 cells. SQSTM1 is a protein that functions as an autophagy receptor shuttling ubiquitinated proteins for autophagosomal mediated degradation. VP treatment caused the disappearance of the autophagy cargo receptor SQSTM1 at the expected molecular weight. VP was recently reported to cause cross-linking of SQSTM1 [41] and in fact, we observed that VP treatment caused a concentration-dependent accumulation of high-MW (> 62 kDa) SQSTM1 that was concomitant with loss of SQSTM1 at the expected MW (Additional file 3: Figure S3A). Such an effect of VP on protein oligomerization was recently reported for STAT3 and mTOR [36, 42]. Treatment of HCT116 and AA-HCT116 with VP induced protein oligomerization of mTOR and GRP78 also in our hands (Additional file 3: Figure S3B, C).

We reasoned that disturbance of SQSTM1 function might alter the dynamics of the UPS in AA-HCT116 cells. Utilizing the RNA-seq data, we could identify a clear differential expression of UPS-related genes in AA-HCT116 cells, suggesting that changes in the UPS could be responsible for the increased sensitivity of AA-HCT116 cells to VP treatment (Fig. 7b). Gene ontology (GO) analysis further supported this observation, revealing enrichment of several UPS related gene ontology terms (Additional file 3: Figure S3D). As observed for Bortezomib (BZ), a known proteasome inhibitor, VP treatment also induced a concentration-dependent accumulation of poly-ubiquitinated proteins and concurrent decrease in the amount of free ubiquitin (Fig. 7c), which normally indicates defective proteasomal degradation and/or deubiquitination activity. Interestingly, VP seems to induce the appearance of polyubiquitin complexes with MW higher than those induced by Bortezomib, suggesting some differences in the mechanism of action of these two drugs. To investigate this possibility, we used Suc LLVY-AMC, a fluorogenic substrate for measuring the chymotrypsin-like peptidase activity of the 20S proteasome. HCT116 and AA-HCT116 cells were treated with 1 and 5 µM VP, with BZ used as positive control. As shown in Fig. 7d–e, while the proteolytic activity of the proteasome was strongly reduced by treatment with BZ, no major effects were observed on either of the cell lines treated with VP. Consistent with this observation, VP did not affect degradation of the proteasome substrate UbG76 V-YFP in the MelJuSo reporter cell line, indicating that proteasomal degradation is fully functional in VP-treated cells (Fig. 7f). Moreover, unlike BZ, VP does not affect the expression of cyclin-dependent kinase inhibitor CDKN1A, a protein whose turnover is regulated by proteasomal degradation (Fig. 7g). Due to the light-dependent effects of VP on cell viability, we assessed the effects of light exposure during the treatment and protein extraction steps. As shown in Additional file 3: Figure S3E, the presence of high MW polyubiquitinated proteins was observed when all experimental procedures were conducted in presence of light. In contrast no polyubiquitinated proteins were observed when all procedures were conducted in complete darkness. Taken together, these experiments show that although VP induced the accumulation of high MW ubiquitinated proteins, this effect is light dependent and not due to inhibition of proteasomal activity.

## Discussion

Tumor acidosis has been acknowledged as an important feature of solid tumors that can dramatically alter pathobiology of the disease [8, 10]. Although it has been known that acidosis also negatively modulates drug sensitivity, few studies have aimed to identify agents with preferential activity towards cancer cells cultured in acidic conditions. Standard 2D cell culture has long been applied for the identification of compounds with activities on either proliferation or cell survival in preclinical settings. Conventional culture conditions imply the use of media with high glucose content, non-physiological oxygen pressure and alkaline pH set at 7.4. Overall, such conditions are poorly representative of the complex physiological environment that cancer cells experience *in vivo* and hence drug screening performed in these simplified conditions might overestimate the potential clinical efficacy of identified compounds. With the aim to partially overcome such limitations, we developed and characterized HCT116 cells chronically adapted to low pH culture conditions. To our knowledge, the characterization of the transcriptome of acid adapted cells has thus far been limited. RNAseq analysis was applied to define major differences between parental and adapted cell lines, as summarized in Fig. 3. Although the full characterization and validation of these data was not in the scope of this study, the analysis suggests distinct biological properties of the low pH adapted AA-HCT116 cells, including a slower proliferation rate and an increased expression of several markers implicated in autophagy and cell differentiation. In line with previous reports on other cell types [34, 43], AA-HCT116 cells show upregulation of several autophagy genes. AA-HCT116 cells also upregulate the expression of CD274 (PDL-1), a molecule whose expression on tumor cells mediates immunosuppression by inhibiting the activation of CTL and NK cells. Interestingly, acidosis is known to have a strong immunosuppressive activity, thus modulation of tumor acidosis has been suggested as a mechanism to improve immune therapy [44–46].

An initial pilot screen with clinically used anticancer drugs showed that nearly all drugs had a lower cytotoxicity on AA-HCT116 as compared to parental HCT116 cells, confirming that cells chronically adapted to acidosis are resistant to many chemotherapeutics. Interestingly, Everolimus was recently reported to selectively target melanoma cells adapted to acidic pH [47] and in our study the activity of Everolimus and Sorafenib was similar in the two cell lines.

These findings prompted us to use systematic screening to identify compounds with cytotoxic activity also against acid-adapted colon carcinoma cells, leading to the identification of VP as an interesting hit, with equipotent activity also on the adapted cell line. VP has been safely used as a photosensitizer in photodynamic therapy (PDT) of acute macular degeneration and it is under investigation in PDT of pancreatic cancer [48]. Recently, it was suggested that VP specifically targets Yes-associated protein 1 (YAP1), a transcriptional coactivator in the Hippo signaling pathway [37]. Based on this report, VP has been used as a pharmacological tool to study the protumorigenic activity of YAP1 in several cancer models [49–51]. Moreover, some studies have also suggested that VP inhibits YAP activity in the absence of light activation [39, 52]. However, a very recent study has shown that VP demonstrate activity against cancer cell lines that is independent of YAP1 and instead dependent on the ability to induce high MW protein oligomers, in particular STAT3 and SQSTM1 [36]. In fact, VP was recently shown to induce cross-linking of SQSTM1 [41]. In line with these studies, we observed that treatment with VP induces the accumulation of protein oligomers, including SQSTM1, GRP78 and mTOR.

Here, we extend these studies to demonstrate that the activity of VP towards cancer cells is pH-dependent and light-dependent and associated with protein cross-linking and alterations of polyubiquitinated proteins. The exact mechanism by which VP induces such a phenomenon is still unclear. However, we could exclude any involvement of the proteasome since we did not observe any inhibition of proteasomal function upon VP treatment using three different methods. Importantly, we observed that treatment of cells and protein extraction in complete darkness totally abolished the effects of VP on the observed accumulation of poly-ubiquitinated proteins, suggesting that extreme care should be taken in experimental studies addressing the mechanism of action of this drug, as recently pointed out by Kostantinou and colleagues [53]. In fact, contrary to many studies where exposure to ambient light is not specifically addressed in experimental procedures, these authors present strong experimental evidence that cross-linked oligomers and formation of high MW complexes is mostly a light dependent mechanism. In line with this, we observed that the effects of VP on cell viability was also light-dependent since exposure of VP to ambient light for 30 min before treatment greatly increased VP effects in both HCT116 and AA-HCT116 cells.

Although the scope of this study was not to unravel the molecular mechanism by which VP affects cancer cell viability, we observed that VP has a preferential activity towards colon cancer cells grown in acidic conditions. This might be simply related to the higher intracellular availability of VP in both AA-HCT116 and parental HCT116 cells transiently exposed to acidic medium. This behavior may be predicted by the acidic pKa of the compound, which likely facilitates the uptake and intracellular availability.

In conclusion, our study describes the phenotypic and transcriptomic profile of acid adapted colon cancer cells. We used these cells to screen a library of FDA-approved compounds and identified Verteporfin as a hit compound preferentially targeting acidic cancer cells. Given that VP (Visudyne^®^) is already available for therapeutic applications, its use as anticancer agent might be easily translated into clinical oncology for specific cancer types.

## Conclusions

Tumor acidosis is an important factor underlying poor efficacy of anticancer drugs. We have characterized cancer cells chronically adapted to acidosis and used them as a tool to screen for drugs, leading to the identification of Verteporfin as an effective drug preferentially targeting acidic cells. To our knowledge this is the first study assessing the relevance of tumor acidosis in a drug screening perspective.

## Additional files

**Additional file 1: Figure S1.** A volcano plot summarizing the results from the differential expression analysis. The five genes with the highest fold change in either direction are highlighted in the plot. Dotted red vertical lines represent a fold change of 2.

**Additional file 2: Figure S2.** Viability assay of 10 hit compounds. The effects of 10 hit compounds on cell viability was measured in HCT116, AA-HCT116 and RPE1 cells. Data from three different experiments are shown.

**Additional file 3: Figure S3.** (A) HCT116 cells were treated with VP for the indicated time points and the expression of SQSTM1 of different MW was assessed by WB. (B) HCT116 and AA-HCT116 cells were treated VP and the expression of mTOR was analysed by WB. Cells were also untreated (C) or treated with EBSS (S). (C) HCT116 and AAHCT116 cells were treated VP and the expression of GRP78 was analysed by WB. (D) Analysis of UPS-related GO terms and associated P values. (E) HCT116 cells were treated with Bortezomib or Verteporfin for 4 h combining light (L) and darkness (D) during treatment of the cells and protein extraction. The accumulation of polyubiquitinated proteins was analysed by WB.
