# Supplementary figures and images for: A drug screening assay on cancer cells chronically adapted to acidosis
Source: Cancer Cell Int. 2018 Sep 25;18:147. doi: 10.1186/s12935-018-0645-5 (PMC6156858; doi:10.1186/s12935-018-0645-5)

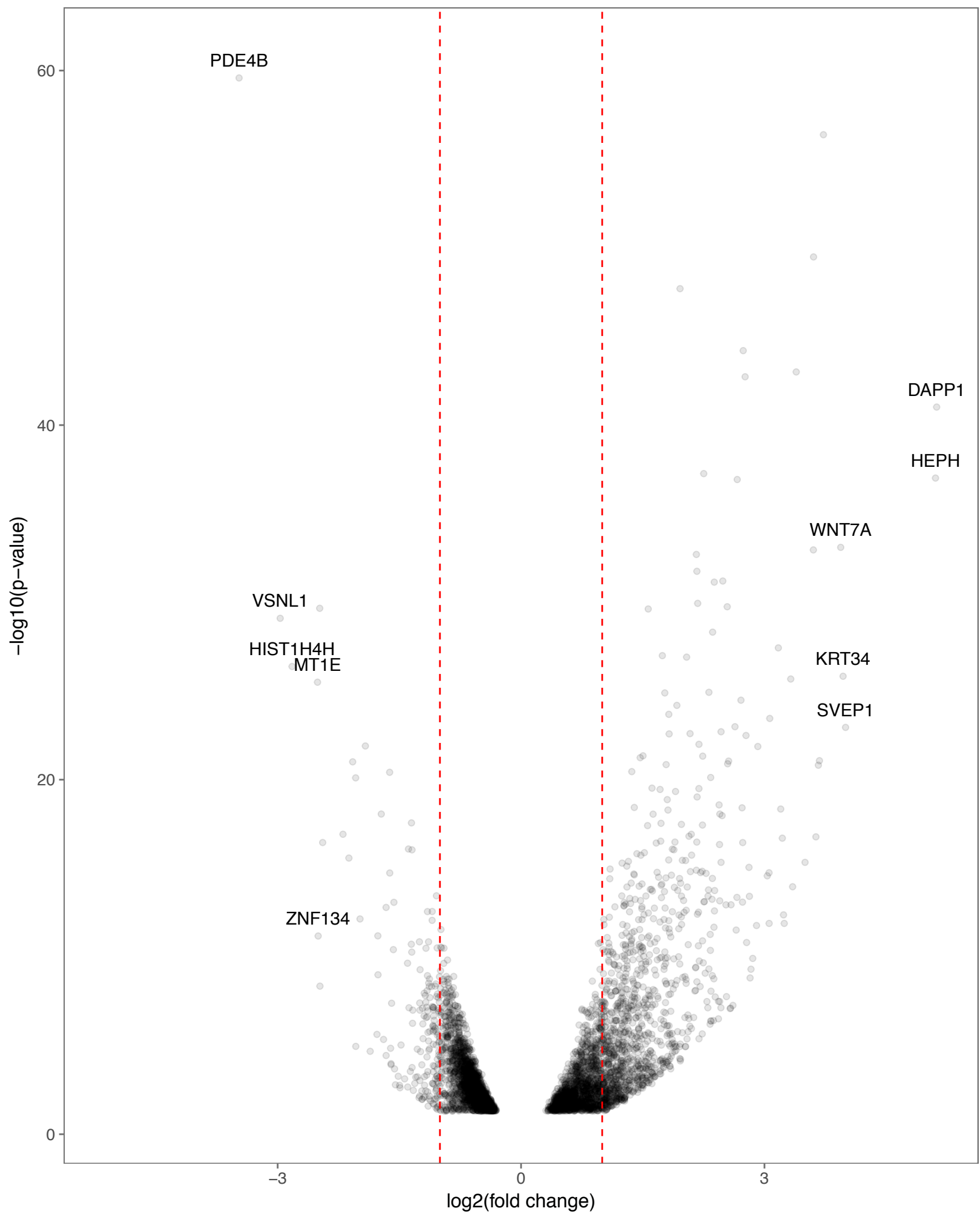

Supplement: Supplementary file 1 — Additional file 1: Figure S1. A volcano plot summarizing the results from the differential expression analysis. The five genes with the highest fold change in either direction are highlighted in the plot. Dotted red vertical lines represent a fold change of 2. [file 12935_2018_645_MOESM1_ESM.pdf]

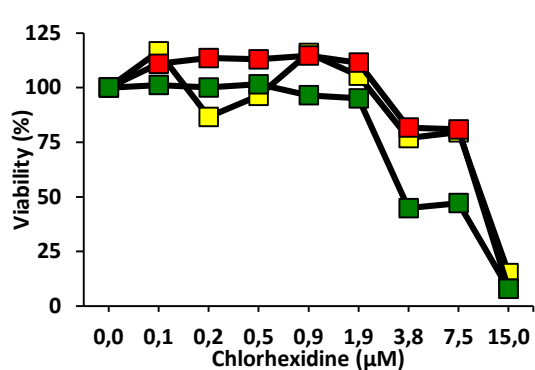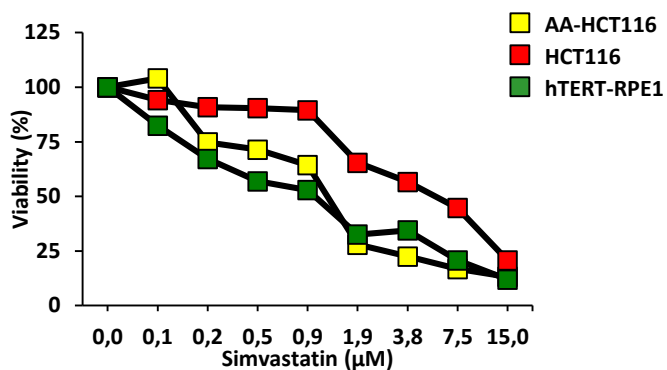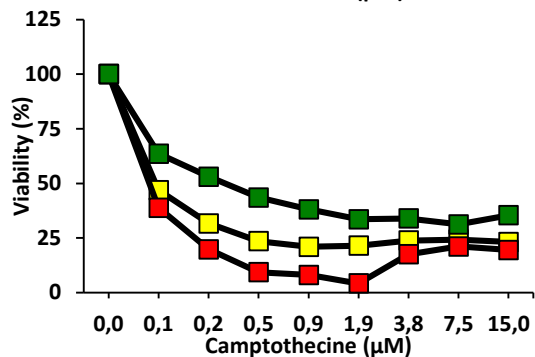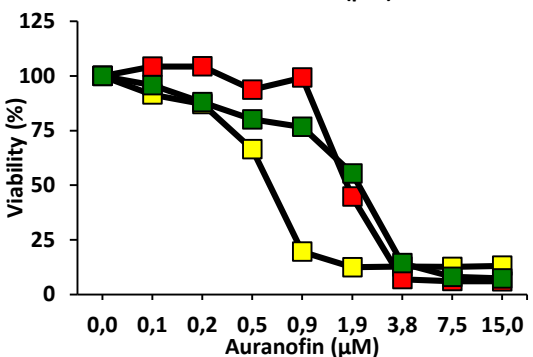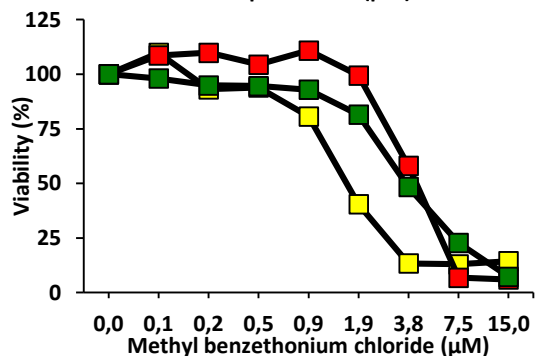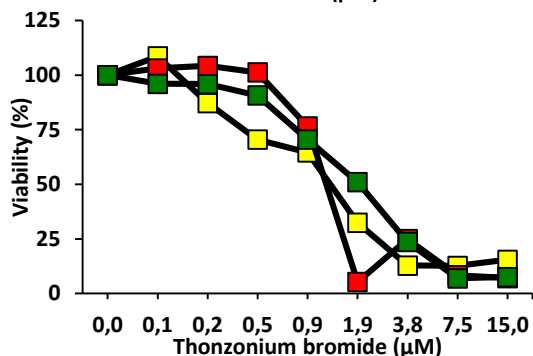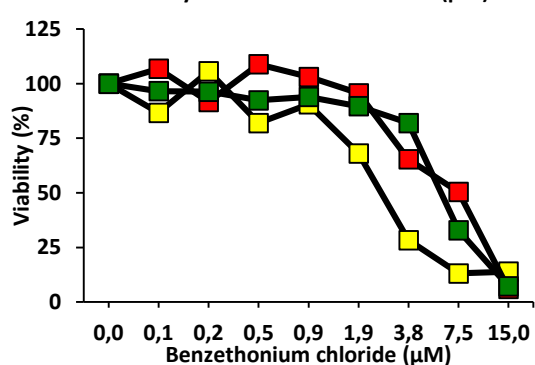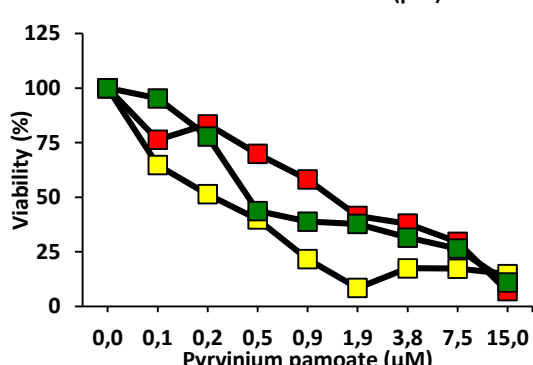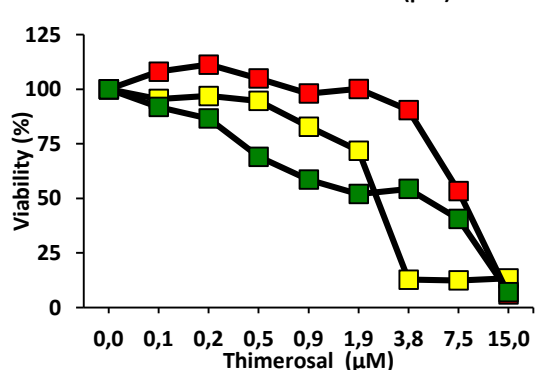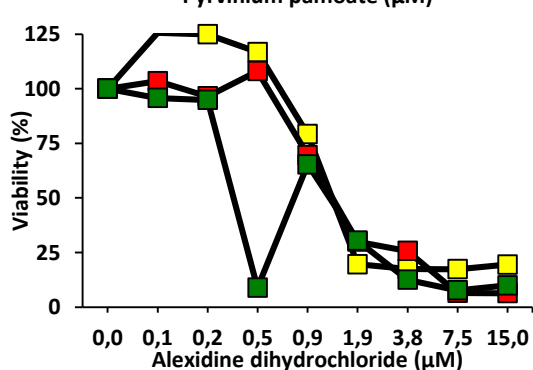

Supplement: Supplementary file 2 — Additional file 2: Figure S2. Viability assay of 10 hit compounds. The effects of 10 hit compounds on cell viability was measured in HCT116, AA-HCT116 and RPE1 cells. Data from three different experiments are shown. [file 12935_2018_645_MOESM2_ESM.pdf]

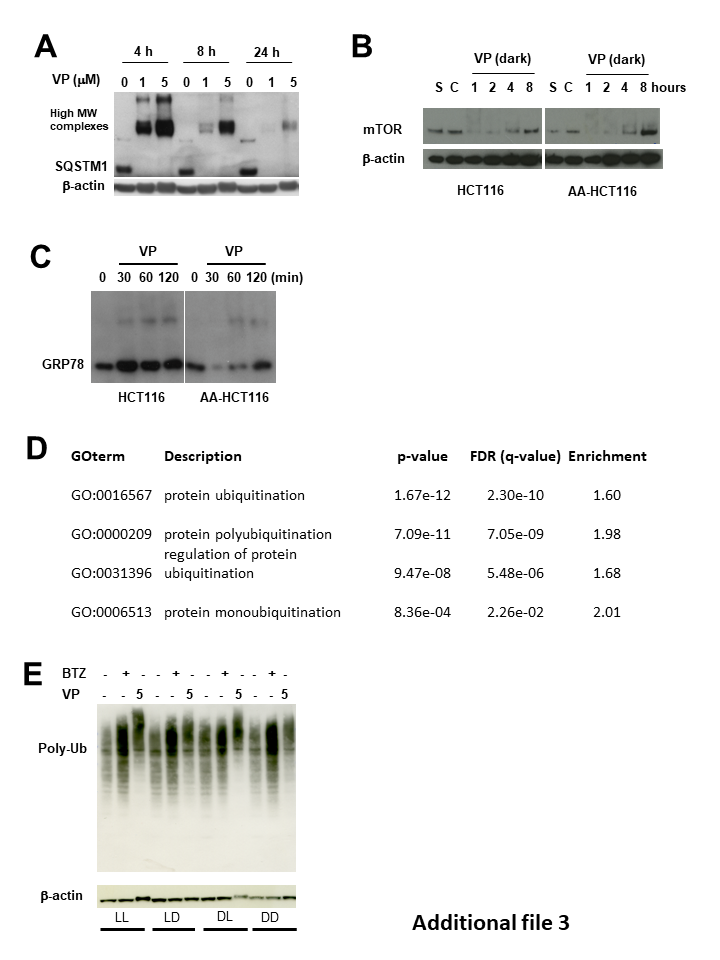

Supplement: Supplementary file 3 — Additional file 3: Figure S3. (A) HCT116 cells were treated with VP for the indicated time points and the expression of SQSTM1 of different MW was assessed by WB. (B) HCT116 and AA-HCT116 cells were treated VP and the expression of mTOR was analysed by WB. Cells were also untreated (C) or treated with EBSS (S). (C) HCT116 and AAHCT116 cells were treated VP and the expression of GRP78 was analysed by WB. (D) Analysis of UPS-related GO terms and associated P values. (E) HCT116 cells were treated with Bortezomib or Verteporfin for 4 h combining light (L) and darkness (D) during treatment of the cells and protein extraction. The accumulation of polyubiquitinated proteins was analysed by WB. [file 12935_2018_645_MOESM3_ESM.png]
